# Newborn Screening for SCD in the USA and Canada

**DOI:** 10.3390/ijns4040036

**Published:** 2018-11-26

**Authors:** Nura El-Haj, Carolyn C. Hoppe

**Affiliations:** Department of Hematology-Oncology, UCSF Benioff Children’s Hospital Oakland, Oakland, CA 94609, USA

**Keywords:** hemoglobinopathies, newborn screening, methods, review

## Abstract

Sickle cell disease (SCD) encompasses a group of inherited red cell disorders characterized by an abnormal hemoglobin, Hb S. The most common forms of SCD in the United States and Canada are identified through universal newborn screening (NBS) programs. Now carried out in all fifty U.S. states and 8 Canadian provinces, NBS for SCD represents one of the major public health advances in North America. The current status of NBS programs for hemoglobinopathies and the screening techniques employed in many regions worldwide reflect in large part the U.S. and Canadian experiences. Although the structure, screening algorithms and laboratory procedures, as well as reporting and follow up, vary between NBS programs, the overall workflow is similar. The current review summarized the historical background, current approaches, and methods used to screen newborns for SCD in the United States and Canada.

## 1. Introduction

Sickle cell disease (SCD) refers to a clinically heterogeneous group of disorders characterized by a structurally abnormal hemoglobin, hemoglobin S (Hb S), inherited in either a homozygous fashion (Hb SS) or in combination with other Hb variants (e.g., Hb SC, Hb SD, Hb S/O-Arab) or a beta thalassemia mutation (Hb S/beta thalassemia).

Under hypoxic conditions, Hb S polymerizes causing red blood cells to become rigid and deformable [1]. These sickled red blood cells adhere to the vascular endothelium, as well as circulating blood cells, leading to vaso-occlusion and impaired tissue oxygenation [2]. Repeated sickling also damages the red blood cell membrane, resulting in a chronic hemolytic anemia.

Elevated levels of fetal hemoglobin (Hb F) normally present at birth prevent Hb S polymerization and hemolysis and protect affected newborns from complications. As Hb F becomes replaced by Hb S in the first few months of life infants with sickle cell disease (SCD) become at risk for life-threatening complications associated with sickling and hemolysis. These complications, including infection and acute splenic sequestration, are associated with increased morbidity and mortality in the first five years of life [3].

In the USA and Canada, the rationale for newborn hemoglobinopathy screening is based on the benefit provided by penicillin prophylaxis against life-threatening pneumococcal infection in infants with SCD [4]. Since implementation of universal NBS for SCD in the United States, mortality has decreased by 50% in affected children ages 1 to 4 years, and the overall life expectancy has increased from a median of 14.3 years to between 42 and 53 years in males, and between 46 to 58.5 years in females [5]. Additional benefits of NBS include prompt clinical intervention for infection or splenic sequestration episodes, and early education of caretakers about the signs and symptoms of illness in infancy and early childhood [6]. Early parent education on assessment of spleen size reduced mortality due to splenic sequestration by nearly 10-fold in an observational study [7]. Moreover, in a 7-year follow up study, NBS was found to be most effective in reducing mortality when coupled with comprehensive medical care and parent education [5,8].In addition to the public health impact on affected infants, NBS carries the added benefit of identifying at-risk couples, providing the opportunity for genetic counseling regarding options for future pregnancies.

## 2. Epidemiology

SCD affects over 300,000 newborns per year worldwide [9]. Although hemoglobin disorders are most prevalent in sub-Saharan Africa, throughout Asia, the Middle East, and around the Mediterranean, population migration from these regions has changed the demographic landscape in North America, where the carrier rates for SCD and other hemoglobin disorders have increased in recent years [10]. Contemporary national data regarding the overall prevalence of sickle cell disease in the United States is lacking. However, data derived from the U.S. Census and NBS programs estimates a sickle cell carrier frequency of 8–10% in African-Americans and 0.6% in Hispanics, respectively, and an overall disease prevalence of 100,000 Americans [11]. 

Using national birth cohort data spanning the 20-year period from 1991–2010, Therrell et al. reported an overall annual birth prevalence of 1:1941 across the USA [12]. Although the absolute number of affected births is lower in Canada, annual birth prevalence rates of SCD in provinces with large multi-ethnic populations, such as Ontario and Quebec, are similar to those in the USA [13]

In Canada, an estimated 3000 to 5000 individuals are living with SCD, the majority of whom reside in Ontario and Quebec. As in the USA, the estimated annual birth incidence of SCD varies geographically with reported estimates of 1:17,721 births in British Columbia, 1:5650 births in Ontario and 1:1852 births in Quebec with the highest rates of 1:2800 births observed in populations of African, Middle Eastern, and Mediterranean descent [14].

## 3. History of NBS for SCD

**Figure 1:**
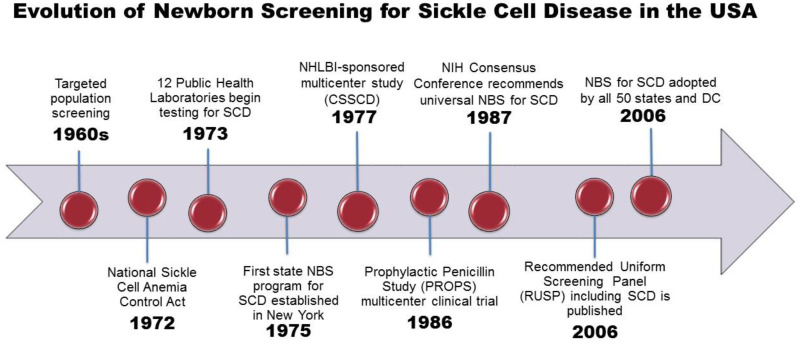
SCD includes Hb SS, Hb S/beta thalassemia and Hb SC genotypes. Abbreviations: ACMG, American College of Medical Genetics; CSSCD, Cooperative Study of Sickle Cell Disease; HHS, Health and Human Services; HRSA, Health Resources and Services Administration; NBS, newborn screening; NHLBI, National Heart, Lung and Blood Institute; NIH, National Institutes of Health; RUSP, Recommended Uniform Screening Panel.

The evolution of NBS for SCD, from the initial discovery of a laboratory test to detect sickle hemoglobin to the development of a complex state-based public health program, has been previously described in detail [15]. An overview of the landmark events leading to NBS for SCD is shown in Figure 1. In the USA, population screening for SCD began in the late 1960′s in response to mounting political pressure by African American advocacy groups. Only a few states performed testing for SCD in newborns and only on a selective basis. 

With increasing recognition of SCD as a significant public health issue, Congress passed the National Sickle Cell Anemia Control Act in 1972, which gave authority to establish education, screening, testing, counseling, research, and treatment programs [16]. At the same time, dried blood spots were introduced as an effective method to collect and test blood samples from newborns [17]. By 1973, 12 state public health laboratories had adopted some form of sickle cell screening program and the first statewide NBS program for SCD was established in New York in 1975 [18].

As the first federal program to support a genetic disease, The National Sickle Cell Disease Program funded over 250 general screening programs, 41 sickle cell centers and clinics, as well as 69 research grants and contracts with numerous locally supported screening, education, and counseling clinics [19]. A centralized Hemoglobinopathy Reference Laboratory was also created at the U.S. Centers for Disease Control (CDC) serving as a national resource to assist states in testing for SCD and other hemoglobinopathies, and to provide external proficiency testing and continuing education [20]. After the CDC Hemoglobinopathy Reference Laboratory’s closure in 1993, screening and confirmation for all hemoglobin disorders was left to the individual state programs and the proficiency testing program was subsequently transitioned to the Laboratory Quality Assurance Program.

Despite national recognition and federal funding for NBS, states outside of New York, were slow to follow in implementing universal NBS [15,21]. By 1983 only 3 states, New York, Colorado, Texas, had added hemoglobinopathies to the screening panel. The landmark Prophylactic Penicillin Study (PROPS) multicenter clinical trial demonstrating the lifesaving benefit of penicillin prophylaxis against pneumococcal infection in infants and young children with SCD, provided the impetus and justification for universal NBS in the USA [4]. Following publication of this study, the National Institutes of Health convened a consensus conference that resulted in unanimous support for universal NBS for SCD, as mandated by state law [22]. Regionally-centered laboratory services were also recommended in order to improve efficiency and minimize potential errors.

Automated dried blood spot (DBS) punching facilitated expanded screening by significantly reducing the sample preparation time and effort. U.S. and Canadian programs were the first to apply computerized data management to NBS in the 1980s. In 1993, the Agency for Health Care Policy and Research (AHCPR) concluded that NBS for SCD combined with comprehensive health care could significantly reduce infant morbidity and mortality rates, and the agency recommended universal screening rather than targeted screening based on race [23].

By 1999, all but nine states in the USA had implemented universal NBS for SCD. However, there was still no national process to ensure uniformity among states in quality of testing, interpretation of results, or collection of outcome data. In response, the Health Resources and Services Administration commissioned the American College of Medical Genetics to gather expert opinion for a consensus document outlining a core panel of conditions, minimum standards, policies, and procedures for NBS programs nationally [24]. Three SCD genotypes (SS, S/beta thalassemia, and SC) were included in the recommended core panel of state-mandated screening conditions. All other hemoglobinopathies were listed as recommended secondary targets for optional screening. By 2006, all 50 states, the District of Columbia, and many U.S. territories had adopted universal NBS for SCD [15].

Although all NBS programs in the US currently screen for the most common forms of SCD (HbSS, Hb SC, and Hb S/β thalassemia), there is wide variability across states with respect to screening, reporting, and referral of other hemoglobinopathies identified in the course of screening for the core panel conditions. Some states screen for a number of secondary conditions, such as Hb SD, that are concomitantly detected by the screening test methods. A few states have expanded the core screening panel to include clinically relevant non-sickling hemoglobin disorders, such as beta thalassemia and Hb H disease, but most states consider these as secondary screening targets and will refer newborns with a screening test showing a Hb F-only pattern or elevated Bart’s hemoglobin for definitive molecular testing [25]. Hemoglobin H disease, an alpha thalassemia disorder prevalent in Southeast Asian, Middle Eastern, and Mediterranean populations, has been given consideration for inclusion as a core condition on the Recommended Uniform Screening Panel (RUSP) RUSP, but lacks sufficient evidence to meet selection criteria [26]

All states require newborn screening, and with the exception of two NBS programs, state statutes that govern screening do not require parental consent. Another 13 states require that parents be informed about NBS prior to testing. All states, except one, allow parents to refuse NBS on religious or personal grounds [27].

Unlike the U.S. where NBS for SCD has been a consideration since 1975, with complete coverage by 2006, screening in Canada was only formalized in one program in 2006, with others slowly coming on board. Targeted screening for SCD began in Quebec in 1988 [13]. The Committee for Development of Newborn Screening for Sickle Cell Disease in Ontario followed in 1989 by launching initiatives and lobbying for universal NBS in Canada. Universal NBS was first implemented in 2006 in Ontario, followed by British Columbia and Yukon in 2009. In 2013, Quebec piloted a universal screening program based on the results from the earlier targeted screening program in Montreal and ultimately implemented a province-wide universal NBS in 2016 [28]. Universal SCD screening is now carried out in 7 Canadian provinces (British Columbia, Ontario, Quebec, New Brunswick, Nova Scotia, Yukon, and Prince Edward Island). Inclusion of SCD on the core NBS panel in Alberta is under consideration pending available funds and service capacity [28].

With no national NBS policy in either the USA or Canada, individual NBS programs screen for a variable number of conditions beyond the recommended core panel, and employ different methods and procedures to carry out screening. Government funding and political support for NBS also vary by region in both countries. Similar to the state governed NBS programs in the USA, universal NBS is under provincial jurisdiction in Canada. Whereas NBS for a recommended core panel of conditions, including SCD, is mandated by individual states in the USA, NBS is considered standard of care in Canada [29]. Moreover, there is no formal province-wide mechanism to document consent, and health care providers are responsible for giving sufficient information to allow parents to make informed decisions, and for documenting parental consent or refusal in the infant’s medical record and/or a signed form indicating parent refusal [27].

## 4. Components of Newborn Screening for SCD

Although logistical aspects, testing methods, and referral policies vary across NBS programs in the United States and Canada, the overarching system for NBS is the same and is comprised of six parts: (1) Education, (2) Screening, (3) Short-Term Follow-Up (STFU), (4) Diagnosis, (5) Management, and (6) Evaluation and Long-Term Follow-Up (LTFU) [30]. NBS programs in both countries follow a similar workflow using newborn DBS specimens, which are tested in specialized screening laboratories and linked to clinical follow-up programs for confirmatory testing and referral to subspecialty comprehensive care [29]. In keeping with national guidelines, state NBS programs in the United States have developed policy and procedure manuals following uniform standards for the performance and documentation of all NBS testing [31].

## 5. Screening

### 5.1. Specimen Collection

NBS programs in the United States and Canada have incorporated SCD into the existing laboratory algorithms, and initial screening is now performed in conjunction with testing for other selected congenital disorders. Both countries perform screening using blood spots collected by heel-prick between 24 to 72 h of age, or prior to discharge from the hospital or birthing facility. The timing of collection for repeat blood specimens varies by state. Almost one-third of states require collection of a second sample at age 1–2 weeks [32]. For infants transfused prior to newborn screening, a repeat specimen is recommended between 90 to 120 days after transfusion, unless DNA testing is part of the NBS protocol.

### 5.2. Specimen Submission

Dried blood spot specimens are sent to a laboratory that is designated by the state or territory within 24–72 h after collection to avoid hemoglobin degradation from prolonged storage and exposure to heat and humidity [33]. Quality checks are performed and the data from the DBS card are entered into a database. Testing is performed within 72 h, typically on the same day the specimen is received in the laboratory.

### 5.3. Testing Methods

Current methodologies recommended by The Agency for Health Care Policy and Research (AHCPR) for initial and second-tier screening include isoelectric focusing (IEF), high performance liquid chromatography (HPLC), and capillary electrophoresis, which are more sensitive and specific than alkaline and acid gel electrophoresis [34]. Less common screening techniques that are continuing to evolve include tandem mass spectrometry (MS/MS) and various molecular methods [35].

Most NBS programs still use a two-tiered approach, wherein the initial test is followed by a complementary method, such as IEF, HPLC, CE, or dual citrate and cellulose acetate electrophoresis [20]. Alternatively, some NBS laboratories use a modified protocol to improve resolution of the initial test method as the second-tier method. Most NBS laboratories in the US and Canada use HPLC and/or IEF to screen for SCD.

As of 2015, an equal number of NBS laboratories participating in the CDC newborn screen quality assurance proficiency testing program used either HPLC or IEF as the primary screening method [20]. Although HPLC and IEF are highly sensitive and specific, results and interpretation can be confounded by extreme prematurity, previous blood transfusion, or degradation of hemoglobin on the DBS [31].

The increasing availability of automated, reliable, and relatively inexpensive molecular technologies has expanded the use of DNA-based methods for confirmation or definitive diagnosis of NBS results. Molecular techniques can be used either as a second-tier screening method or as a confirmatory test. DNA analysis is performed for second-tier testing in three states (New York, Texas, and Washington). In California, DNA testing on a second specimen is included as part of the screening protocol. In Canada, molecular testing may be requested for NBS results indicating a presumptive β-thalassemia or other genetic variants. Samples are referred to a centralized specialty laboratory for testing at no cost [36].

## 6. Short Term Follow up

### 6.1. Primary Screening Results Reporting

As with the provision of NBS laboratory services, follow up and education are also defined by individual NBS programs in both the USA and Canada. Short-term follow up begins with communication of the presumptive positive screening results to the submitting hospital, physician of record, and/or designated NBS follow-up program.

In Canada, all screen-positive test results are reported to the primary care provider, as well as to the provincial Regional Treatment Centers responsible for tracking all presumptive positive cases [36]. The primary care provider (PCP) is given recommendations for the necessary follow up, including initiation of penicillin prophylaxis and is responsible for arranging confirmatory testing on a second liquid blood sample “recall specimen.” 

A few state NBS programs have incorporated a web-based screening information system allowing follow-up staff at regional coordinating centers and health care providers to directly access the NBS results [37]. This system has been shown to facilitate tracking of positive results and timely enrollment of infants into a comprehensive treatment program.

### 6.2. Confirmatory Testing

All abnormal or unusual screening results require follow-up of the presumptive screen-positive result through a confirmatory testing protocol to verify the screening result. Policies and practices for confirmatory testing of hemoglobinopathies vary by state. Even in cases where molecular testing on the initial NBS specimen has confirmed the likelihood of a disorder, additional testing is necessary to verify the specimen identification, determine the type of SCD, and to prove or disprove an initial result indicating a presumptive hemoglobinopathy of potential clinical significance. A screening result report of “Other” or “Unknown” hemoglobin variant(s) (often abbreviated as “Hb V”) usually indicates the presence of a hemoglobin variant for which the proper comparison to a validated control has not been made. In Canada, confirmatory testing of SCD follows a similar pathway, performed on a liquid blood sample, in contracted reference laboratories, using similar laboratory techniques, either HPLC and/or IEF.

In both the US and Canada, abnormal screening results are reported to a dedicated follow-up agency, sickle cell treatment center or provider to ensure that the newborn and the family have access to appropriate follow-up care. Each US state and Canadian province is responsible for directing its follow-up program state involving NBS program staff, primary care providers, hematologists, and genetic counselors [38]

In the USA, the processes and algorithms for notification and follow-up of newborns with confirmed SCD often place the primary care physician as the initial “medical home,” which ideally includes specialists with expertise in SCD, as well as families and community-based organizations working in partnership to obtain the comprehensive services needed. Many states have long-standing contracts with academic SCD and thalassemia centers to provide follow-up services [39]. However, the extent of the comprehensive services provided by the “medical home” is variable and often limited by a lack of resources, competing priorities or incomplete understanding of child health quality measures and care [39].

## 7. Long-term Follow-up

Long term follow-up is part of the quality assurance component of the NBS system, specifically the evaluation of the effectiveness of the NBS follow-up program in ensuring access to treatment and preventive care throughout the lifespan [40]. In both Canada and the US, treatment and follow-up services are delivered through programs that are either government sponsored, private or public-private collaborations [29]. Currently, all states are required to follow established standards for laboratory practices [41]. All laboratories that perform testing for state NBS programs voluntarily participate in the CDC’s Newborn Screening Quality Assurance Program (NSQAP) to verify the accuracy of the screening tests performed.

However, many state programs fall short in tracking the long-term data from comprehensive care centers and specialists, and in evaluating the outcomes. The absence of standard case definitions across states has also been identified as a barrier to aggregate data for comparative analyses. To address this challenge, a HRSA-funded data repository, the Newborn Screening Technical assistance and Evaluation Program (NewSTEPs), was created to facilitate data collection using a uniform nomenclature [42]. These case definitions have been piloted, but not yet validated. State NBS programs are encouraged to utilize the case definitions and to enter case data into the repository [42].

The Health Resources and Services Administration (HRSA)-sponsored Registry and Surveillance System for Hemoglobinopathies (RuSH) is another example of a data resource for evaluating the quality of NBS follow-up by linking multiple data sources across seven states for population-level surveillance of newborns and individuals with SCD [43].

## 8. Challenges of NBS for SCD

Newborn screening has improved the prognosis for individuals with SCD through early intervention measures, such as penicillin prophylaxis and immunization and early education. However, there remain ongoing challenges with regards to the timely follow-up and implementation of comprehensive care [44]. Wide variation in reporting of screening results by states has led to delays in early intervention [38]. Other states have reported gaps in compliance with early medical intervention, parental education, and the provision of comprehensive health services.

As state NBS programs have different systems for capturing data, the use of different case definitions to evaluate the burden and birth prevalence of SCD has been challenging. Federally-sponsored capacity building for long term follow-up data capture is now a focus across many states, using resources to support coordinated data collection and tracking of cohorts of individuals with SCD to evaluate the long term outcomes [45]. While systems linking state NBS programs using common data definitions are in development, there is currently no mechanism linking NBS systems with clinical data systems, nor is there a standardized approach to assess the impact of NBS on health outcomes in children.

Patient registries using medical record linkage with NBS programs are being explored to facilitate the transfer and exchange of information between the NBS program and clinical providers [46]. Health information technology (e.g., electronic medical record exchanges and interoperability standards) provides a tool for consistent data collection, current care practices, and identifying gaps. The costs associated with building this capacity for long term follow-up remains a major challenge. Moreover, the decline in reimbursement for services from public insurance programs has further limited the ability to provide adequate clinical care and education to affected patients [23].

Only a few NBS programs have established activities focused on policy development and system change [47]. Despite the recently published NHLBI evidence-based clinical standards of care for SCD, the available preventative and therapeutic interventions are not reaching the affected individuals [48]. A framework for widespread application of the NHLBI guidelines, using implementation science methods, has been proposed to close the “quality gap” [47].

## 9. Conclusion

The history of NBS for SCD in the USA and Canada spans many years beginning with the recognition of SCD as a significant public health issue, and the identification of hemoglobinopathies from the same dried blood spot used to screen for other congenital disorders. Many of the screening tests that have become part of routine screening worldwide were initially developed in the U.S. and Canadian laboratories, including hemoglobinopathy testing. In both countries, the components of NBS include a screening process incorporating specimen collection, submission, and a two-tiered testing approach using complementary methods to identify a hemoglobinopathy. However, NBS programs in both countries use different methods of testing and reporting results to public health programs, hospitals, and individual providers, with wide variation in the content and format. Although the implementation of NBS for SCD has led to improved outcomes in children, few studies have evaluated the long-term health outcomes in the growing population of adults with SCD. Efforts to standardize nomenclature and collection of outcomes data through the development of linked registries are a first step in achieving the long-term follow-up goal to ensure appropriate delivery of health care to individuals with SCD identified by the NBS.
